# Comprehensive Evaluation of Bacillus Calmette–Guérin therapy in Non-muscle invasive bladder cancer Egyptian patients: a retrospective cohort study

**DOI:** 10.1186/s43046-026-00340-3

**Published:** 2026-01-26

**Authors:** Riham M. Karkeet, Mohamed M. Sayed-Ahmed, Shahenda Ghaly, Mayar Farouk, Nourhan Yasser, Nada Hany, Malak Atta, Ahmed M. Amer, Ahmed Abdelbary

**Affiliations:** 1 Cairo University, Giza, Egypt; 2 NewGiza University, Giza, Egypt; 3 School of Medicine, Badya University, Badya city, Giza Egypt

**Keywords:** Non-Muscle Invasive Bladder Cancer (NMIBC), Alkaline phosphatase, Urea, Creatinine, Bacille Calmette-Guerin (BCG), Side effects, Performance status, Diabetes Mellitus, Pathological parameters

## Abstract

**Background:**

Bladder cancer ranks among the most prevalent genitourinary malignancies globally, demanding comprehensive management strategies. Bacille Calmette-Guerin (BCG) therapy, a standard for Non-Muscle Invasive Bladder Cancer (NMIBC), is integral to treatment, yet debates persist regarding its optimal application.

**Aim:**

This research aims to elucidate potential correlations and implications for BCG immunotherapy. Moreover, explores the impact of BCG-induced localized and systemic side effects on NMIBC outcome, addressing the need for a nuanced understanding of its therapeutic and adverse effects.

**Patients and methods:**

Patients’ data was sourced from NMIBC patient files at The National Cancer Institute—Cairo University, comprising a total of 150 patients’ files. Demographic and pathological data, BCG side effects, performance status (PS), recurrences and progression were recorded. The European Organization for Research and Treatment of Cancer (EORTC) score guided risk assessment was employed. ALP, urea and creatinine levels were also assessed at baseline and after the 1st, 3rd, and 6th dose of BCG to establish correlations with the number of BCG doses administered.

**Results:**

An association emerges, indicating that the dosage of BCG therapy significantly influences recurrence-free survival. Remarkably, patients receiving more than 10 doses showed significantly lower recurrence-free survival (RFS) compared to those administered fewer than 10 doses (*p* = 0.001). Univariate analysis showed that patients who received ≥ 10 BCG doses had worse RFS compared to those who received < 10 BCG doses (Hazard Ratio (HR): 4 [95% confidence interval (CI): 2.4–6.7]), *p* < 0.001. The study reveals a remarkable correlation between the elevation of ALP, urea, and creatinine levels and the number of BCG doses administered. On multivariate analysis, the only independent factor that significantly affects the RFS was number of doses of BCG. Patients who received ≥ 10 doses had worse RFS compared to those who received < 10 doses (HR: 3.8 [95%CI: 1.8–8.1]), *p* = 0.001. Cardiac comorbidity negatively impacted the PFS with *P*-value (0.016). The only significant pathological parameter impacting OS was grade of tumor, *P*-value (0.025). Moreover, size of tumor influenced RFS, *P*-value (0.001). Clear relation between diabetes mellitus and tumor size was observed; where larger tumor size was more prevalent in diabetic patients and reflected as shorter RFS**.** Significant localized adverse effects included increased frequency of urination, pus in urine, and urgency. Pus in urine doubled the risk of recurrence-free survival (RFS). 6.1% reported vomiting and 23.5% experienced fever. Performance status tended to decline post BCG doses. Patients who had vomiting episodes had worse outcome (*p* = 0.01).

**Conclusion:**

The current findings suggest that cardiac comorbidity, high tumor grade and large size might be evaluated as independent poor prognostic factors for Egyptian NMIBC patients. Performance status decline correlated with BCG therapy; tuberculosis-like symptoms were undetected in the currently studied cohort due to the mass vaccination program implemented in Egypt. The research also highlights the impact of the number of BCG doses and ALP baseline on patients’ outcome, providing valuable insights into the sophisticated relationship between treatment intensity and key biomarkers in NMIBC management.

**Supplementary Information:**

The online version contains supplementary material available at 10.1186/s43046-026-00340-3.

## Introduction

Bladder cancer is the tenth most common cancer worldwide [1]. Despite improved survival rates due to advancements in its management, it remains a substantial worldwide health burden [2]. In Egypt, Bladder cancer is the 2nd most common solid malignancy in males [2]. Egypt had the highest total fatality rate due to bladder cancer in 2020 [3].

The prevalence of bladder cancer rises with age and people over 65 years account for 80% of cases [4]. Greater tobacco use and occupational exposure in men could help clarify the four-fold gender difference in bladder cancer incidence [5, 6]; where males have a higher incidence and prevalence than females [7]. Over the past 26 years, there has been a dramatic change in Egypt's histological bladder cancer profile. Historically, transitional cell carcinoma (TCC) has surpassed squamous cell carcinoma as the most common type of bladder cancer in Egypt; this is mostly because Schistosomiasis has been successfully controlled. The recent increase in TCC cases can be attributed to environmental risks, notably smoking [8, 9].

A transurethral technique is frequently used to remove non-muscle-invasive bladder cancer followed by intravesical treatment including Bacillus Calmette–Guérin (BCG) instillations. An induction of six weekly instillations followed by additional maintenance instillations for up to 36 months is the recommended treatment strategy for high risk NMIBC [10, 11]. The long-term probability of progression and recurrence is decreased in high-risk NMIBC patients who get BCG [12]. It is recommended that patients with NMIBC of moderate risk either have an intravesical induction course of chemotherapy or BCG, followed by maintenance. Patients who failed response to intravesical chemotherapy may benefit from BCG therapy [13]. The therapeutic action of BCG is based on stimulating the immunity to attack tumor cells in the bladder, which causes a complicated inflammatory response that targets tumor cells while sparing healthy urothelial cells [14].

Intravesical BCG might cause localized or systemic side effects ranging in severity from mild to severe; as a result of the targeted immune stimulation and cytokine generation [15]. There is insufficient evidence regarding whether lower number of BCG doses correlates with considerably lower side effects [16]. Monitoring patient response through markers as alkaline phosphatase (ALP), urea, and creatinine can contribute valuable insights into the treatment's impact on overall health, particularly kidney function [17]. Understanding the intricate dynamics between BCG immunotherapy and these biomarkers is crucial for optimizing treatment outcomes and minimizing potential side effects.

Based on literature review, the aim of this study is to identify potential correlations and implications for BCG immunotherapy. Moreover, to test the impact of clinical factors related to the patient and disease on oncological outcomes within an Egyptian NMIBC cohort.

## Patient and methods

This is a descriptive retrospective cohort study. The ethics approval was obtained from the research ethical committee for experimental and clinical studies at School of Pharmacy, New Giza University with number CP-0035. In this study, patients’ data was sourced from NMIBC patient files at The National Cancer Institute-Cairo University's (NCI-CU) Epidemiology and Biostatistics department, comprising a total of 150 patients’ files. The sample size was determined through collaboration with the Epidemiology and Biostatistics department according to the rate of patients flow at the urologic-oncology surgery clinic to include all patients who visited the NCI over the past two years. Patient file selection was based on an inclusion and exclusion criteria, patients were eligible for inclusion in the study if diagnosed with stage 1 non-invasive bladder cancer, receiving intravesical BCG and excluded if presented with an autoimmune disease. Insufficient follow-up data resulted in the exclusion of 18 subjects from the analysis.

Comprehensive demographics, pathological criteria (tumor grade, size, focality), ECOG (Eastern Cooperative Oncology Group) performance status and local/systemic side effects were meticulously recorded. Essential information such as the date of diagnosis, progression, recurrence, last follow-up, and death were documented to facilitate the calculation of recurrence-free survival, progression-free survival, and overall survival.

Additionally, the study took into account the number of BCG doses administered and the frequency of TURB-T (transurethral resection of bladder tumor) to analyze their impact on recurrence-free survival. Patients receive 6 weekly cycles of BCG intravesical treatment after complete TURB-T [15]. The European Organization for Research and Treatment of Cancer (EORTC) score was used for non-muscle invasive bladder cancer patients in order to estimate the likelihood of progression and recurrence across one and five years. Six clinical and pathological criteria are included in the score: tumor grade, tumor size, number of tumors, past bladder cancer recurrence rate, carcinoma in situ and disease stage (T category). Each individual is allocated a score between 0 and 6 for each of these variables [18]. Moreover, patients were stratified according to the EAU classification to define the risk groups who received BCG treatment. All patients who were included in the analysis (132 patient) received a minimum of 5 induction weekly instillations (128 patient received 6 and 4 patients received 5 induction weekly instillations) and 51/85 patients from the high risk group received maintenance BCG therapy. Alkaline phosphatase (ALP) levels were assessed at baseline and after the 1 st, 3rd, and 6th dose of BCG; Supplementary 1. According to the NCI policy; routine laboratory tests were performed with each visit including full kidney and liver functions which where extracted from the files. This investigation aimed to identify any elevation in ALP, urea and creatinine levels and ascertain their associations with BCG therapy. The data was used to calculate the grade of toxicity experienced by each patient to assess severity of side effects post-BCG administration. Status of patients, dead or alive, progression and recurrence status was recorded. Follow-up started at the date of TURBT and the analysis started 3 months post the date of TURBT. Recurrence was considered if detected 3 months after TURBT. Patients who showed recurrence within 3 months of first TURBT were excluded from the analysis.

### Statistical analysis

IBM SPSS Advanced Statistics (Statistical Package for Social Sciences), version 22 (SPSS Inc., Chicago, IL), was used to analyze the data. Numerical data will be described as median and Interquartile range (IQR), as data is not normally distributed due to biological variances. Categorical variables were presented as numbers and percentages. Survival analysis was done using Kaplan–Meier method. Comparison between two survival curves was done using log rank test. Cox regression survival analysis was performed to verify the independent prognostic factors affecting DFS Multivariable Cox proportional regression with stepwise selection was used to verify the prognostic significance. *P*-value ≤ 0.05 will be considered significant and all tests will be 2 tailed.

## Results

**Fig. 1:**
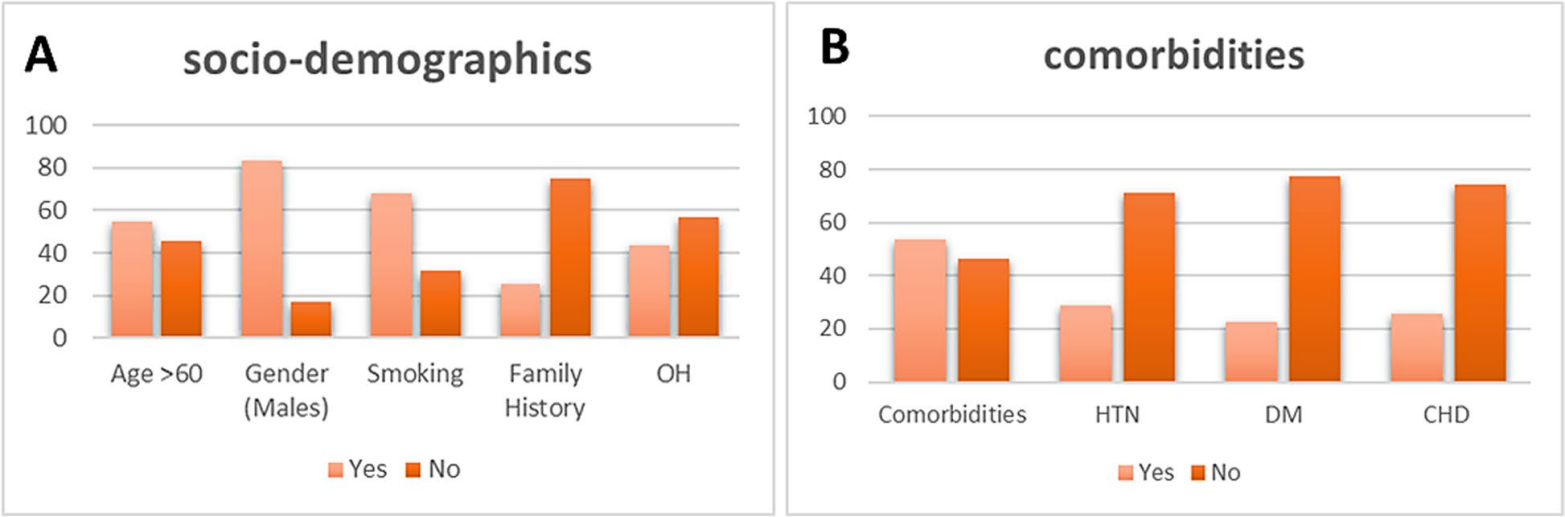
Sociodemographics distribution (**A**) and comorbidities (**B**) among 132 patients with NMIBC; presented as percentages

**Fig. 2:**
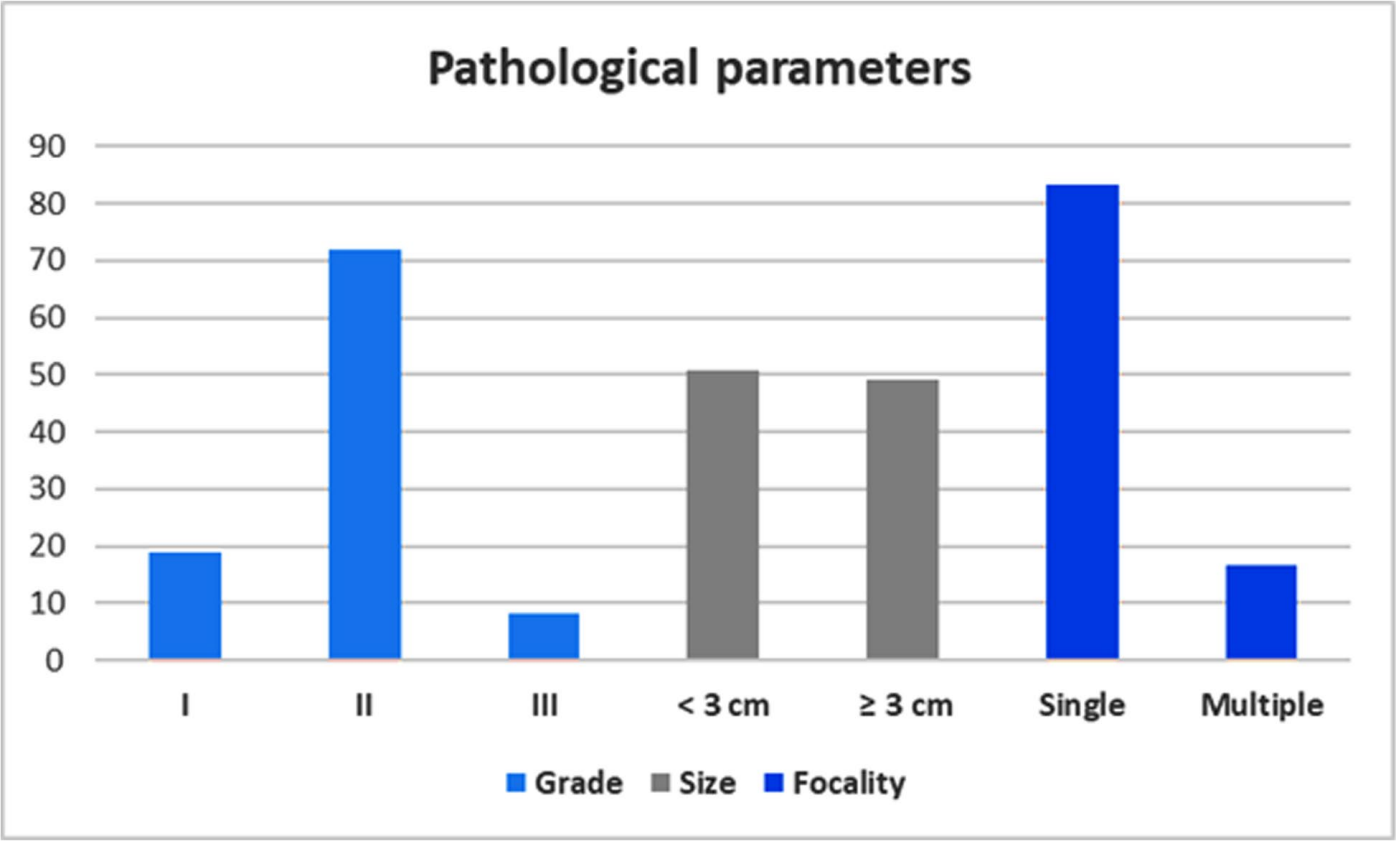
Pathological parameters among 132 patients with NMIBC; presented as percentages

The study included 150 participants; of those 132 met the required criteria. Demographic characteristics of the study cohort shown in (Table 1) provided a comprehensive overview of the participants, encompassing diverse factors influencing health outcomes. The gender distribution was 83.3% males and 16.7% females. The age range spanned from 25 to 91 years, with a median age of 62 years (IQR = 14), reflecting a broad spectrum of adult participants. Notably, observed variations in starting performance status (PS) and current PS scores, highlighting the heterogeneity in participants' health statuses before and after BCG intravesical immunotherapy. Occupational hazards were a pertinent aspect of the current analysis, with 43.2% of patients reporting exposure to specific occupational risks; including farmers, roads or buildings construction and chemicals or ceramic factories. Family history had no significance in the study, as 75% of patients had no familial predisposition to cancer. Additionally, the presence of co-morbidities was diverse; ranging from cardiovascular diseases to diabetes. 53.8% of the studied cohort had co-morbidities, further emphasizing the complexity of the health profile within the study population (Fig. 1). Lastly, 68.2% of patients were smokers. After analyzing the pathological data (presented in Fig. 2), it is evident that the majority of patients had tumor grade 2 and tumor size of ≤ 3 cm with a percent of (72.0%) and (50.8%) respectively. In-addition, single-focality being observed in (83.3%) of the total patient count. Finally, (39.4%) experienced more than one recurrence annually.

**Table 1:** Demographic characteristics

Parameter		Count (*n*=132)	Percent (%)
Gender	Male	110	83.3%
	Female	22	16.7%
Age 60	≤ 60 years	60	45.5%
	> 60 years	72	54.5%
Occupational hazard	No	75	56.8%
	Yes	57	43.2%
Family history	No	99	75.0%
	Yes	33	25.0%
PS start	PS I	128	97.0%
	PS II, III, IV	4	3.0%
PS post	PS I	84	63.6%
	PS II	13	9.8%
	PS III	22	16.7%
	PS IV	13	9.8%
Comorbidities	No	61	46.2%
	Yes	71	53.8%
Hypertension	No	94	71.2%
	Yes	38	28.8%
Diabetes mellitus	No	102	77.3%
	Yes	30	22.7%
Cardiac comorbidity	No	98	74.2%
	Yes	34	25.8%
Smoking	No	42	31.8%
	Yes	90	68.2%
Tumor Grade	Grade 1	25	18.9%
	Grade 2	95	72%
	Grade 3	12	9.1%
Multi-focality	Single	110	83.3%
	Multiple	22	16.7%
Tumor size	≤ 3 cm	67	50.8%
	≥ 3 cm	65	49.2%
Number of recurrence	≤ 1 recurrence in 1 year	80	60.6%
	> 1 recurrence in 1 year	52	39.4%
EAU risk classification	Intermediate risk	47	35.6%
	High risk	83	62.9%
	Very high risk	2	1.5%
BCG therapy	Induction	132	100%
	Maintenance	51 out of 85	60%

**Fig. 3:**
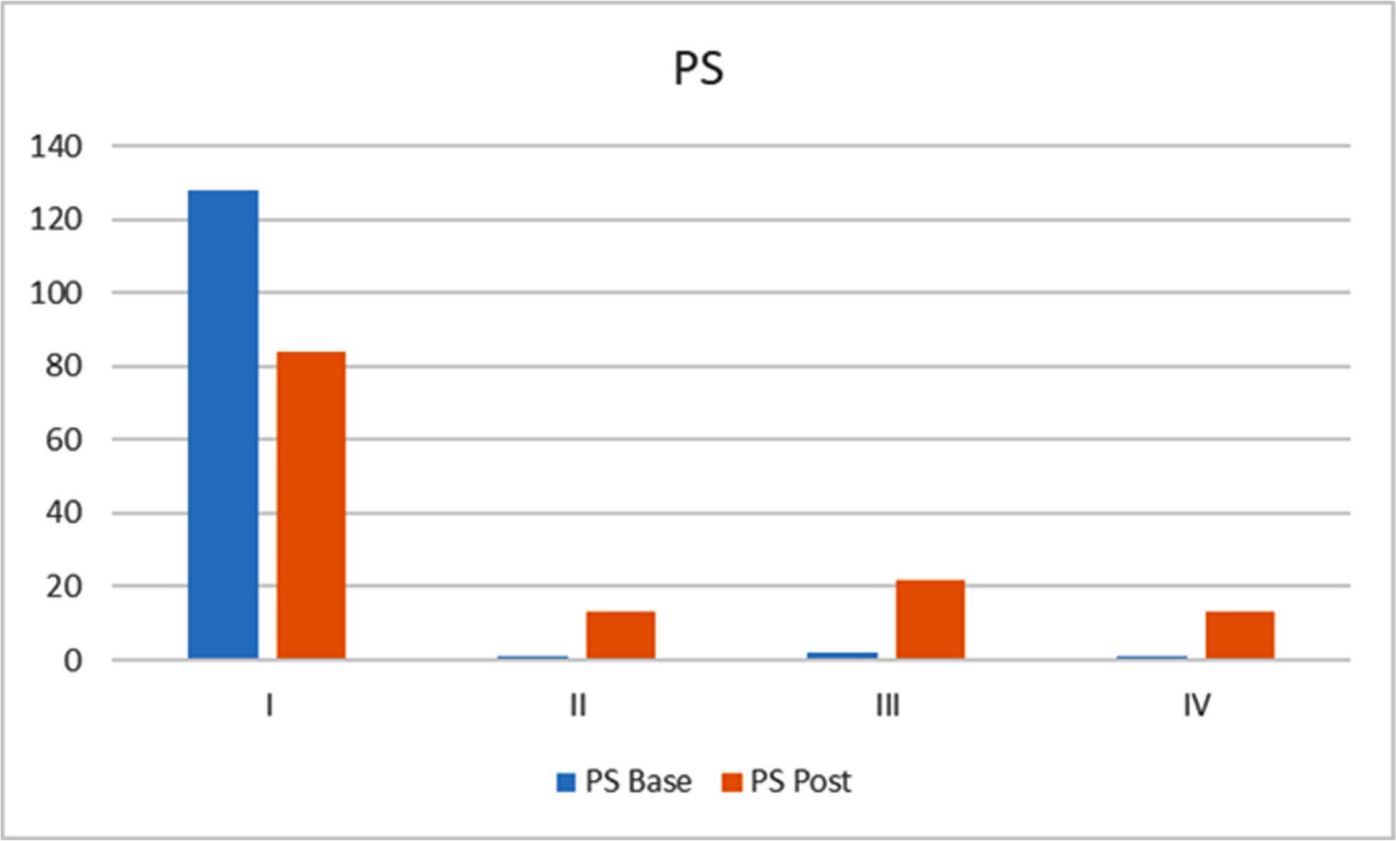
ECGO performance status before and after BCG doses; presented as numbers

Regarding performance status (ECOG**)** presented in Fig. 3, 128 patients (97%) had a starting grade of (ECOG 1), while 4 patients (3%) had grades 2, 3 and 4. After finishing the BCG doses 84 patients (63.6%) had a performance status of 1, 13 patients (9.8%) had a status of 2, 22 patients (16.7%) had a status of 3, and 13 patients (9.8%) had a status of 4.

**Fig. 4:**
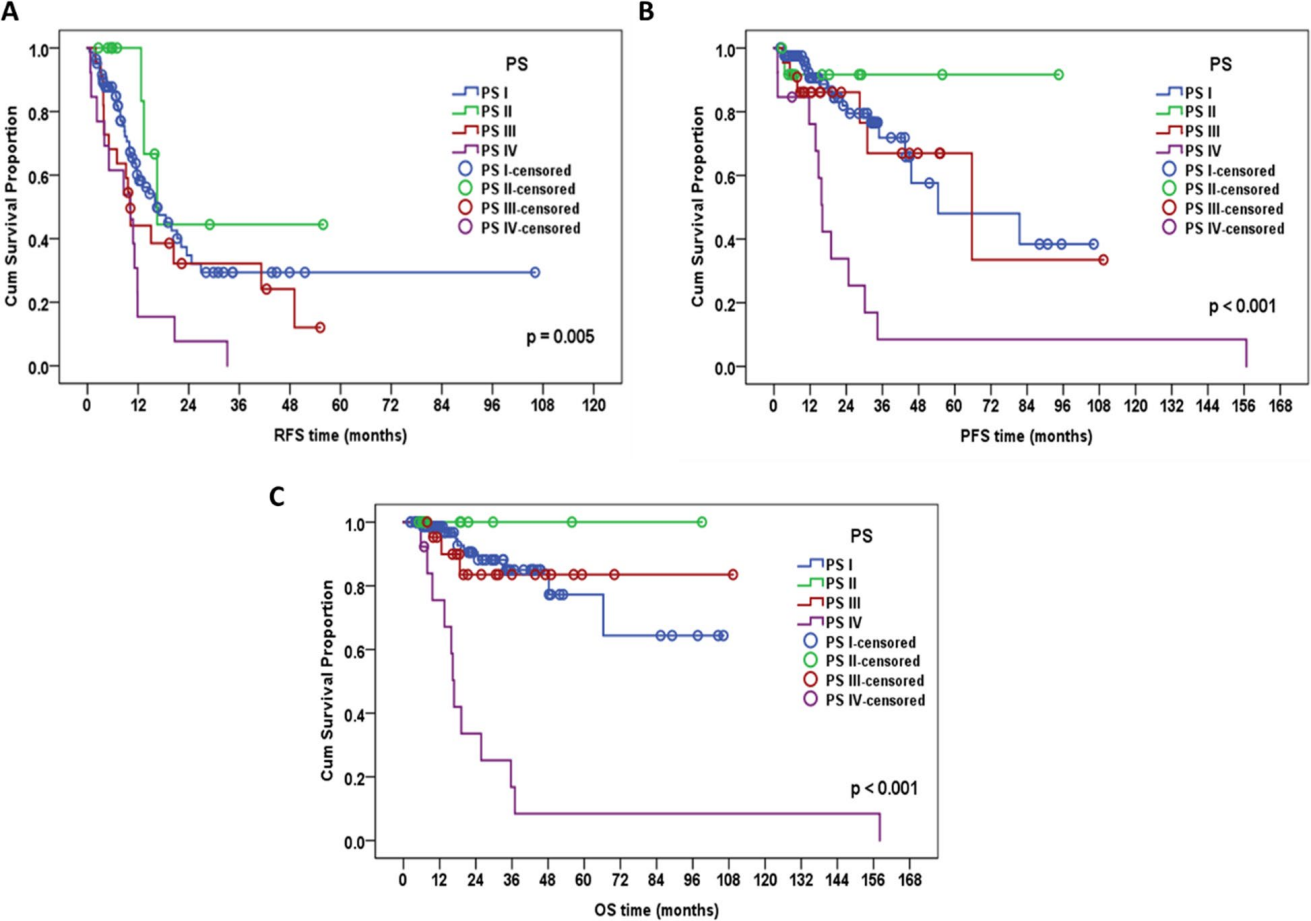
The survival analysis of different PS; A (RFS), B (PFS) and C (OS). Impact of socio-demographic parameters on survival (RFS, PFS and OS) using Kaplan–Meier analysis and Cox proportional hazard model

As depicted in Fig. 4; upon investigating the performance status of the studied cohort it revealed a trend of decline post BCG doses, this was clearly reflected in the survival analysis; where PS4 had a shorter RFS of 10 months, PFS of 16 months and OS of 17 months; compared to PS1 had a longer RFS of 16 months, PFS of 54 months. Univariate analysis PFS using Cox-proportional hazard mode; showed that patients who had PS4 had worse PFS compared to those who had PS1 (HR: 4.74 [95%CI:2.2–10.2), *p* = 0.001. Also, patients who had PS4 had worse OS compared to those who had PS1 (HR: 9.17 [95%CI:3.8–22.3), *p* = 0.001.

**Fig. 5:**
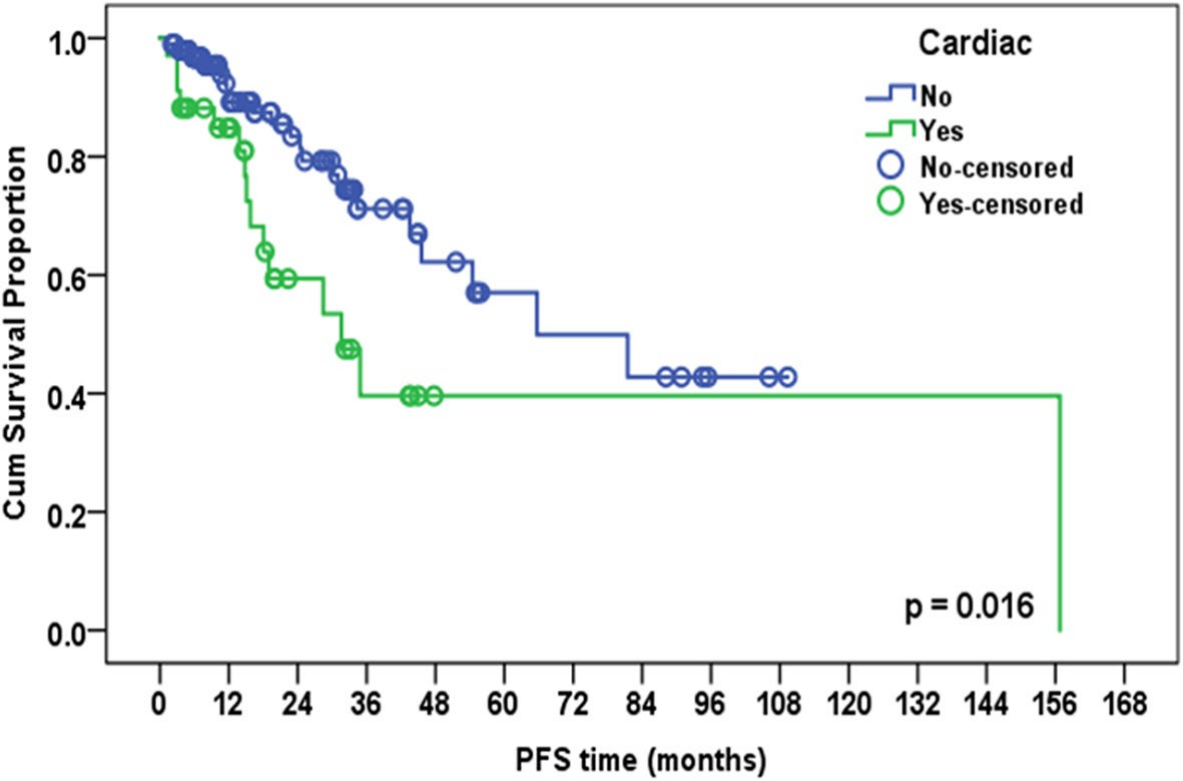
Impact of cardiac comorbidity on PFS. Impact of pathological parameters on survival (RFS, PFS and OS) using Kaplan–Meier analysis and Cox proportional hazard model

All recorded socio-demographics (gender, age, occupational hazard, family history, smoking, comorbidities) did not impact survival. Cardiac comorbidity impacted the PFS, P-value (0.016), where patients with cardiac comorbidity had a shorter PFS of 31.6 months compared to 65.7 months in patients with no cardiac morbidity. Other comorbidities as hypertension (HTN) and diabetes mellitus (DM) did not influence it, (Fig. 5). Univariate analysis of progression free survival (PFS) using Cox-proportional hazard model, confirmed that cardiac comorbidity significantly affected the PFS, (HR: 2.27 [95%CI: 1.14—4.50]), *p* = 0.019. Multivariate analysis revealed that cardiac comorbidity impacted PFS with *P*-value (0.001) (HR: 3.9 [95%CI: 1.79–8.59]).

**Fig. 6:**
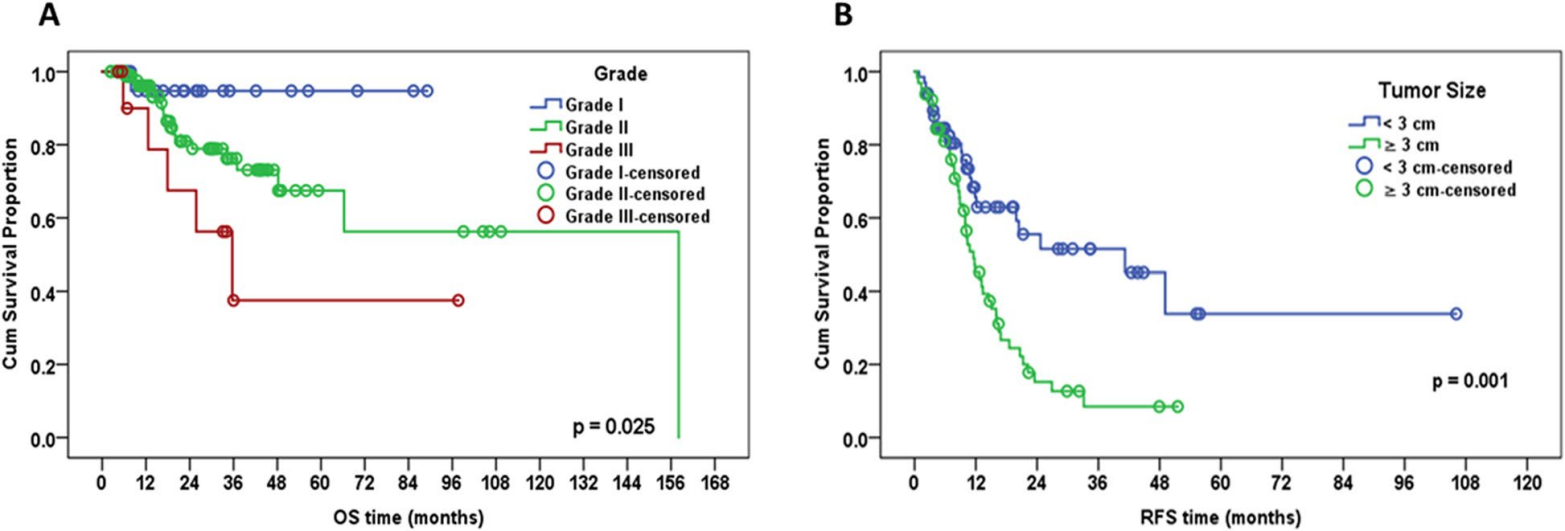
Pathological parameters impact on survival; tumor grade on OS (**A**) and tumor size on RFS (**B**)

The overall survival was impacted by the grade of tumor, *P*-value (0.025) where grade 3 tumors showed a shorter overall survival by 36 months. This was confirmed by the univariate analysis of OS; where it showed that grade of tumor impacted OS with *P*- value (0.032) (HR: 1.72 [95%CI: 1.05–2.83.05.83]), as well as tumor size (≥3 cm versus < 3 cm); *P*-value (0.001) (HR: 2.28 [95%CI: 1.39–3.75.39.75]) (as shown in Fig. 6). Moreover, univariate analysis of PFS showed that Grade (III versus I) affected PFS (HR: 4.4 [95%CI: 1.10–17.76.10.76]), *p* = 0.036. Additionally, size of tumor influenced RFS, *P*-value (0.001); were patients with tumor size ≥ 3 had a shorter RFS of 11.5 months compared to 41 months in patients with tumor size less than three. Other pathological characteristics did not influence the RFS.

**Fig. 7:**
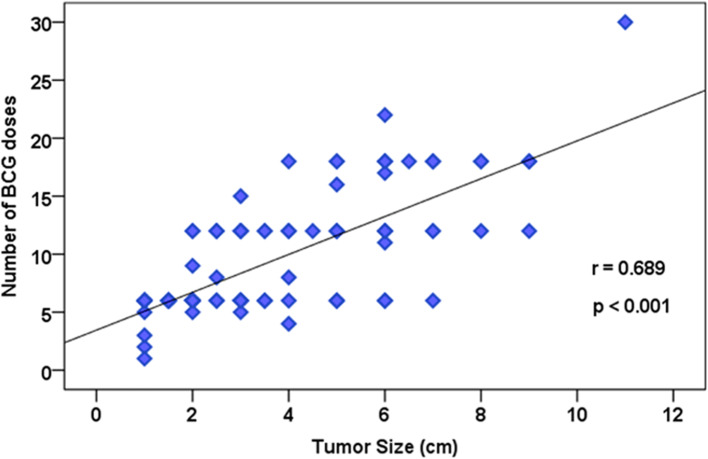
Spearman correlation between number of BCG doses and tumor size

Spearman correlation analysis presented in Fig. 7 revealed a strong positive correlation between number of BCG doses and tumor size, *P*-value (< 0.001).

**Fig. 8:**
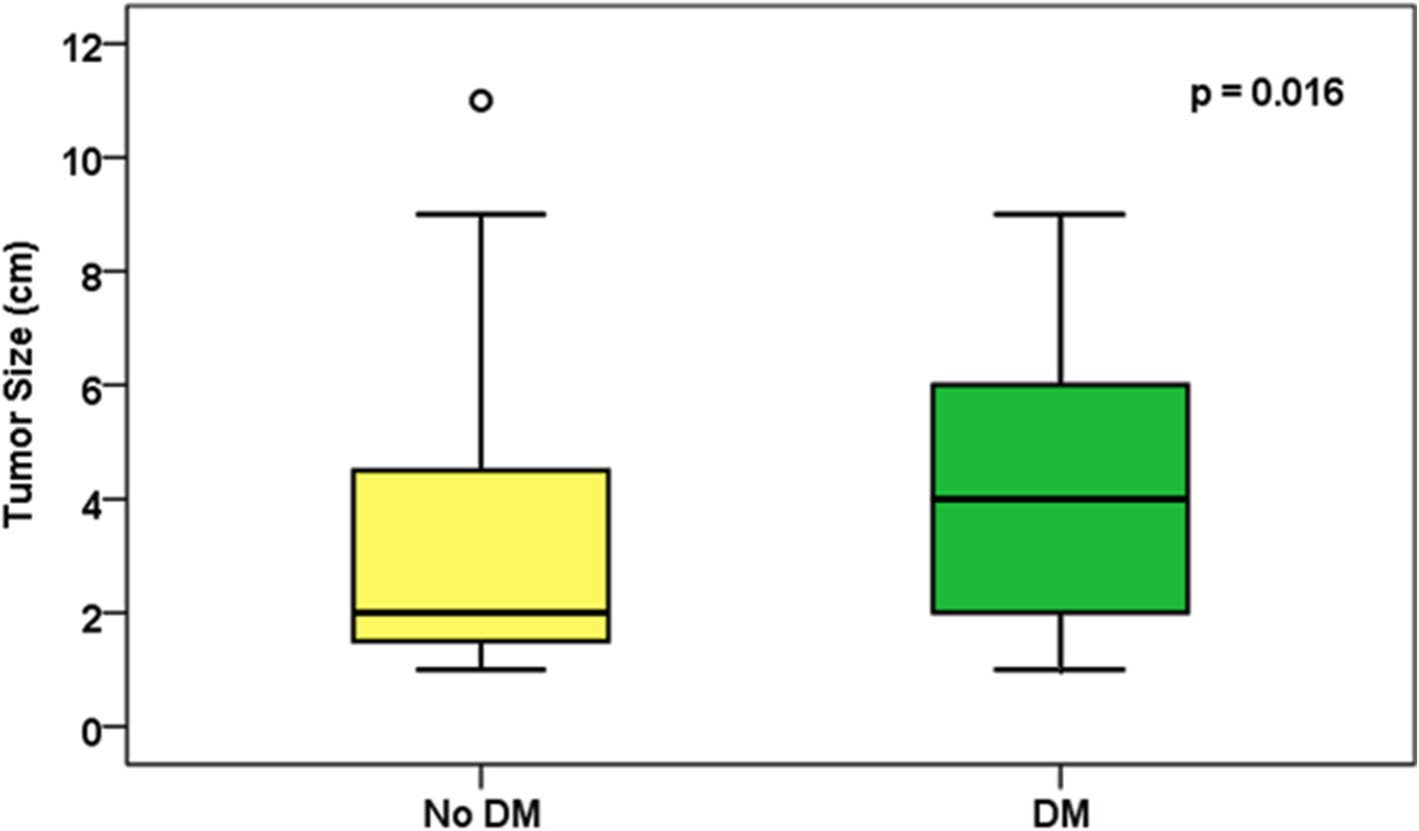
Box-plot relation between tumor size and diabetes mellitus

Diabetics had a significantly larger tumor size compared to non-diabetics (4 cm [1–9 cm] versus 2 cm [1–11 cm] respectively; *p* = 0.016), as illustrated in Fig. 8. Size of tumor influenced RFS, *P*-value (0.001); were patients with tumor size ≥ 3 had a shorter RFS of 11.5 months compared to 41 months in patients with tumor size less than three. Other pathological characteristics did not influence the RFS.

As illustrated in (Table 2), the examination of key biochemical markers and treatment variables within the study cohort, the baseline alkaline phosphatase (ALP) levels indicated a moderate dispersion of values around the central tendency. Sequential readings of ALP at time points 1, 3, and 6 suggested a progressive elevation with each dose. The IQRs corresponding to these readings were respectively highlighting the increasing variability in ALP levels. Urea levels, reflective of renal function, demonstrated a moderately varied distribution. Concurrently, creatinine levels denoted moderate variability. Pertaining to the administered Bacillus Calmette-Guérin (BCG) doses, the median amounted to six, with an associated IQR of six, delineating a diverse range in the therapeutic interventions received by study participants. These refined descriptive statistics lay the foundation for nuanced explorations into the dynamic interplay of variables within the investigative framework.

**Table 2:** Baseline values for serum ALP, urea and creatinine

Parameter	Median	Minimum	Maximum	IQR
Baseline ALP	**81.0**	**24.0**	**281.0**	**38**
ALP1	**88.0**	**27.0**	**291.0**	**32**
ALP3	**110.0**	**36.0**	**704.0**	**45**
ALP6	**137.0**	**32.0**	**1048.0**	**66**
Baseline Urea	**31.0**	**12.0**	**138.0**	**18**
Urea1	**36.0**	**16.0**	**184.0**	**21**
Urea3	**44.0**	**15.0**	**205.0**	**28**
Urea6	**59.0**	**21.0**	**225.0**	**33**
Baseline Creatinine	**1.00**	**0.60**	**10.00**	**0.40**
Creatinine1	**1.10**	**0.60**	**11.50**	**0.45**
Creatinine3	**1.36**	**0.79**	**10.50**	**0.80**
Creatinine6	**1.90**	**0.90**	**10.20**	**1.00**
Number of BCG doses	**6.0**	**1.0**	**30.0**	**6.0**

*ALP* Alkaline Phosphatase, 1: after 1 st dose of BCG, 3: after the 3rd dose of BCG, 6: after the 6th dose of BCG)

**Fig. 9:**
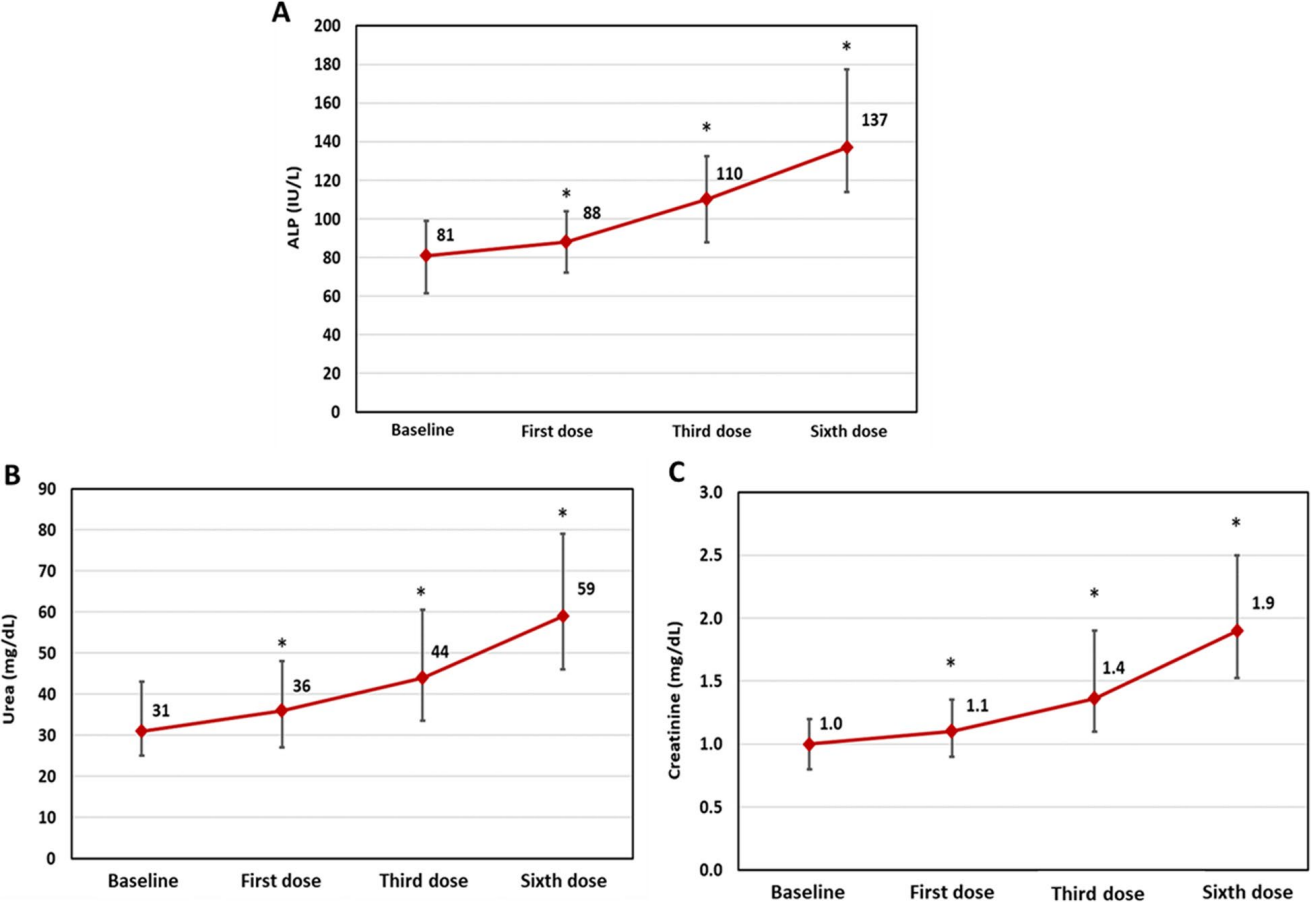
Alkaline phosphatase (**A**), Urea (**B**) and Creatinine (**C**) trend changes in patients with NMIBC over the BCG doses

As demonstrated in Fig. 9, ALP baseline, as well as readings at 1 st, 3rd, and 6th intervals during treatment, consistently demonstrated a discernible elevation over time. This intriguing pattern was mirrored in urea and creatinine levels, suggesting a synchronized physiological response. The results of this study provide an inclusive outline for understanding NMIBC dynamics. The follow-up period was 24 months, the median time to progression was 16 months and the median time to recurrence was 10 months. These findings not only delineate the average timelines, but also emphasize the considerable individual variability in follow-up, disease progression, and recurrence durations within the study cohort.

## BCG number of doses and number of TURT impact on recurrence-free survival

**Fig. 10:**
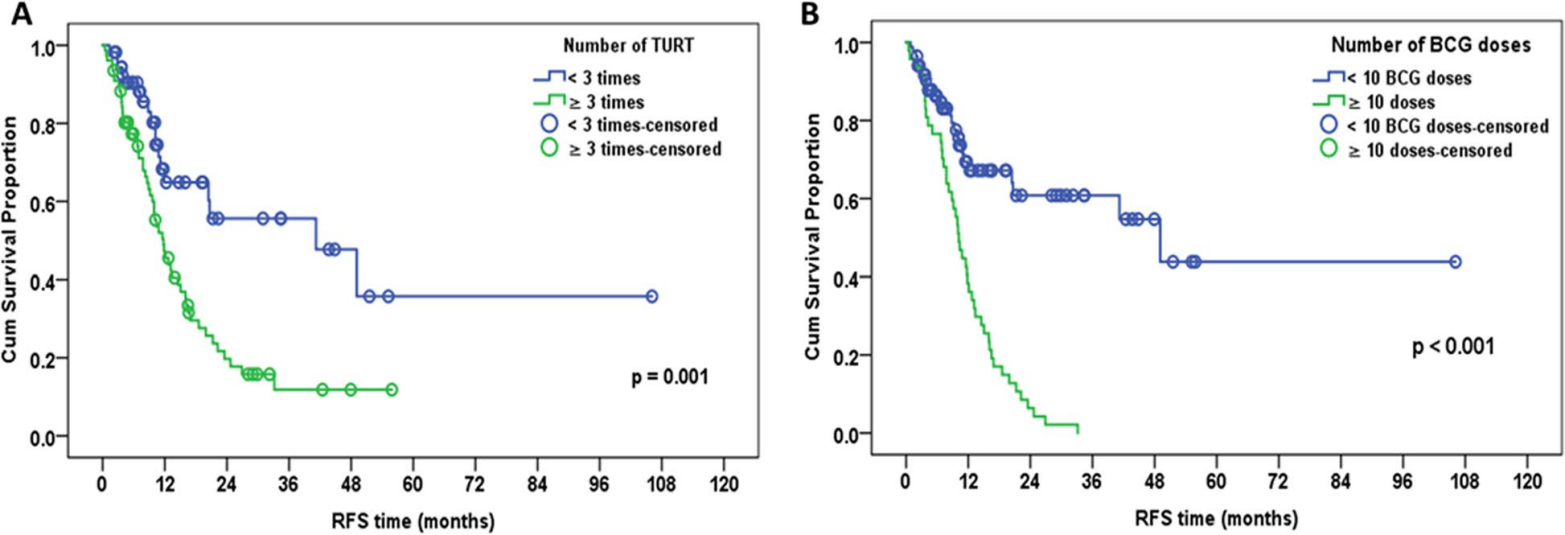
Number of TURT (**A**) and number of BCG (**B**) doses impact on RFS. Predictors of Progression-Free Survival in NMIBC patients taking BCG immunotherapy

This study showed an association between BCG doses and RFS in NMIBC. Remarkably, patients receiving more than 10 doses (induction and maintenance summation) exhibited significantly lower RFS compared to those administered fewer than 10 doses (*p* = 0.001). This statistically significant finding highlights the potential impact of BCG dosage on the clinical outcome. In harmony, patients who had less than 3 TURT recorded a longer recurrence free survival of 41 months compared to 12 months in patients who had three or more TURT, as shown in Fig. 10. Moreover, univariate analysis of RFS using Cox-proportional hazard modle; revealed that patients who had ≥ 3 TURT had worse RFS compared to those who received < 3 TURT (HR: 2.5 [95%CI:1.47–4.32), *p* = 0.001.

The univariate analysis using Cox-proportional hazard model showed that the only independent factor that significantly affect the RFS was number of doses of BCG. Patients who received ≥ 10 doses had worse RFS compared to those who received < 10 doses (HR: 4 [95%CI: 2.4–6.7]), *p* < 0.001. On multivariate analysis of RFS, the only independent factor that significantly affect the RFS was number of doses of BCG. Patients who received ≥ 10 doses had worse RFS compared to those who received < 10 doses (HR: 3.8 [95%CI: 1.8–8.1]), *p* = 0.001.

**Fig. 11:**
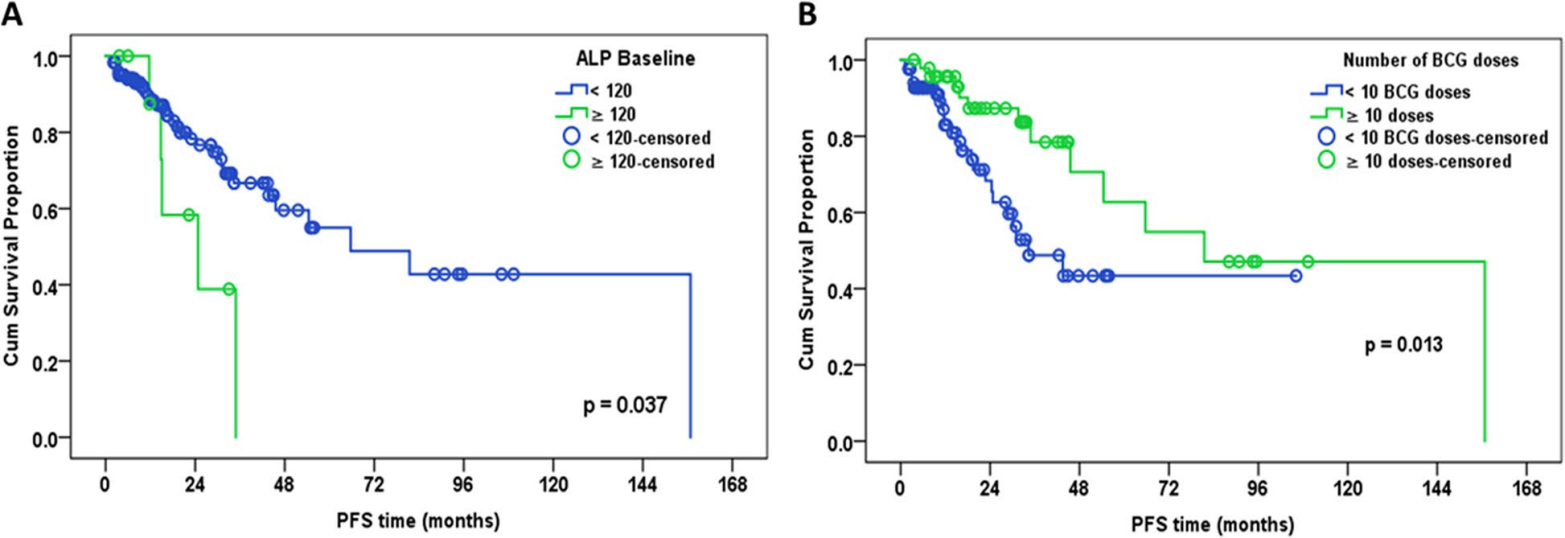
Predictors of progression free survival; (**A**) ALP, (**B**) number of BCG doses

The results indicate that patients with ALP levels above 120 U/L exhibited a significantly shorter PFS compared to those with levels below this threshold. Similarly, the trend persisted at subsequent intervals after the 3rd dose of treatment, Fig. 11A. However, at the 1 st and 6th doses, while the association between ALP levels above 120 U/L and shorter PFS persisted, the chi-square analysis did not reach statistical significance, suggesting a potential divergence in the predictive value of ALP at this specific time point. These findings highlight the importance of monitoring ALP levels in assessing PFS during BCG therapy. On univariate analysis; baseline ALP significantly affect the PFS. Patients who had ≥ 120 U/L had worse PFS compared to those who had < 120 U/L (HR: 2.68 [95%CI: 1.10–7.03]), *p* = 0.045.

This study showed that individuals with a baseline and post-treatment urea level equal to or above 40 mg/dl demonstrated lower PFS compared to those with levels below 40 mg/dl at each time point. Further, individuals with baseline and post-treatment creatinine levels equal to or greater than 1.5 mg/dl exhibited lower PFS than those with levels below 1.5 mg/dl at each time point. Although these observations did not reach statistical significance, the trends suggest potential associations that warrant further exploration.

This study highlighted that individuals receiving BCG doses above 10 demonstrated higher PFS compared to those administered fewer than 10 doses, with *p*-value of 0.013, Fig. 11B. This finding emphasizes the importance of considering BCG dosage as a key factor influencing progression outcomes in NMIBC patients, highlighting its potential implications for treatment strategies and patient care decisions. The univariate analysis revealed that BCG number of doses significantly affects the PFS. Patients who received ≥ 10 doses had better PFS compared to those who received < 10 doses (HR: 2.48 [95%CI: 1.18–5.21]), *p* = 0.016. On multivariate analysis, number of doses of BCG affected the PFS. Patients who received ≥ 10 doses had better PFS compared to those who received < 10 doses (HR: 2.38 [95%CI: 1.10–5.51), *p* = 0.001.

### BCG immunotherapy impact on overall survival in NMIBC

**Fig. 12:**
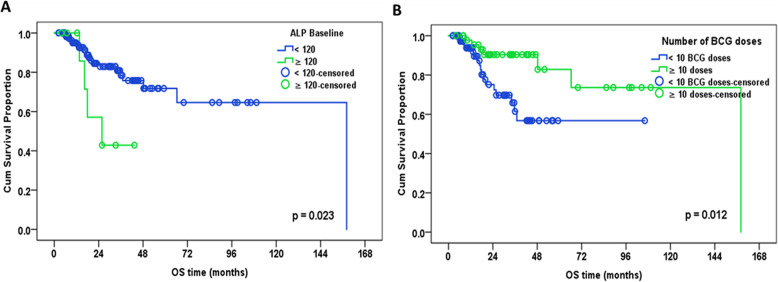
Predictors of OS; (**A**) ALP baseline, (**B**) number of BCG doses

The investigation of ALP levels in NMIBC patients yielded that individuals with a baseline ALP above 120 U/L exhibited lower overall survival compared to those with a baseline ALP below 120 U/L, p-value of 0.023, Fig. 12A. Surprisingly, after the first dose of BCG, ALP levels maintained a similar impact on overall survival as baseline, with a p-value of 0.025. Subsequent doses of BCG further demonstrated intriguing associations, with ALP after the third dose showing a significant p-value of 0.004. However, ALP levels after the sixth BCG dose, did not reach statistical significance. These nuanced findings underscore the complexity of ALP dynamics in NMIBC progression and the potential implications for patient prognosis. The univariate analysis of baseline ALP, ALP1, ALP3 showed significant effect on OS. Patients who had ≥ 120 U/L had worse OS compared to those who had < 120 U/L (HR: 3.3 [95%CI: 1.1–9.9), *p* = 0.032, 0.006.

This study delving into urea levels in NMIBC patients using BCG unveiled that individuals with a urea baseline and post-treatment levels equal to or above 40 mg/dl demonstrated lower overall survival compared to those with levels below 40 mg/dl at each time point. The analysis revealed a compelling result: urea levels after the 3rd dose of BCG showed a significant association with overall survival, indicated by a p-value of 0.036. Patients who had urea level after the 3rd dose of BCG ≥ 40 mg/dl had worse OS compared to those who had < 40 mg/dl (HR: 2.77 [95%CI: 1.03–7.47]), *p* = 0.044. Individuals with a baseline and post-treatment creatinine level equal to or greater than 1.5 mg/dl exhibited lower overall survival compared to those with levels below 1.5 mg/dl at each time point. Creatinine levels after the first and third doses of BCG showed significant associations with OS, with p-values of 0.042 and 0.043, respectively. These significant results emphasize the potential prognostic value of monitoring urea and creatinine levels in predicting overall survival outcomes for NMIBC patients. The univariate analysis revealed that patients who had creatinine level ≥ 1.5 mg/dl had worse OS compared to those who had < 1.5 mg/dl (HR: 2.34 [95%CI: 1.05–5.4]), *p* = 0.048.

Individuals receiving BCG doses above 10 exhibited higher overall survival compared to those administered fewer than 10 doses, and this association reached statistical significance with a p-value of 0.012, Fig. 12B. This finding underscores the potential impact of BCG dosage on the survival outcomes of NMIBC patients, highlighting the importance of dosage considerations in the management and prognostication of NMIBC. On the univariate analysis; number of BCG doses significantly affects the OS. Patients who received ≥ 10 doses had better OS compared to those who received < 10 doses (HR: 3.22 [95%CI: 1.24–8.42]), *p* = 0.017.

**Fig. 13:**
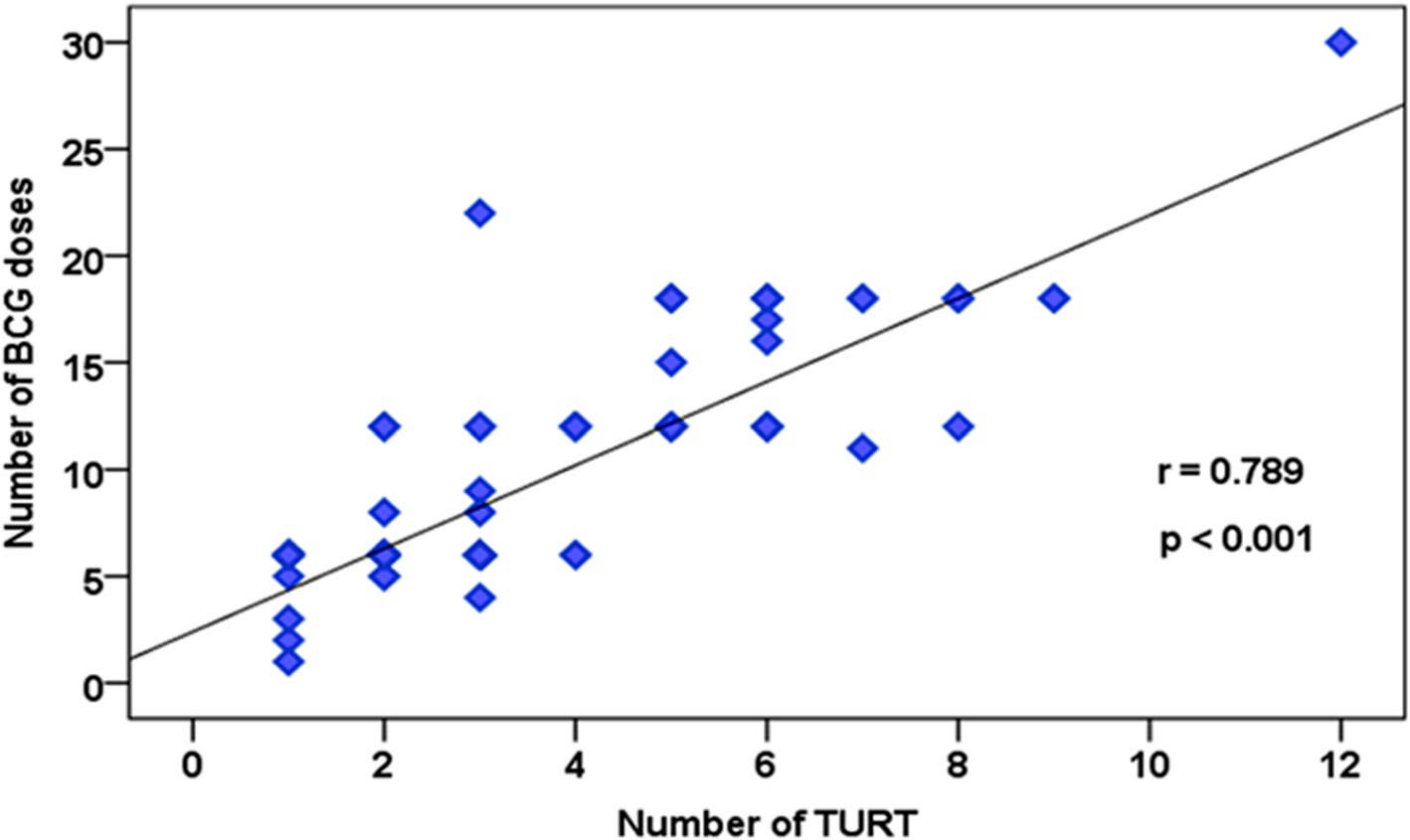
The correlation of the number of BCG doses administered in relation to the number of TURT performed

Spearman correlation analysis presented in Fig. 13 illustrated that assossiation between the number of TURT procedures and the number of BCG doses revealed a p-value of < 0.001; confirming the expected strong relation.

To summarize the impact of ALP, urea, creatinine and number of BCG doses on survival, Table 3 show the numerical values.

**Table 3:** Numeric Insights: Recurrence, Progression, and Overall Survival in BCG immunotherapy in NMIBC

	Recurrence-free survival		Progression-free survival		Overall-free survival	
Baseline ALP	< 120	55.5	< 120	78.3	< 120	84.5
	≥ 120	40.5	≥ 120	58.3	≥ 120	57.1
ALP1	< 120	54.8	< 120	79.6	< 120	84.6
	≥ 120	49.7	≥ 120	58.9	≥ 120	67.5
ALP3	< 120	58.4	< 120	79.4	< 120	87.2
	≥ 120	46.6	≥ 120	71.9	≥ 120	74.0
ALP6	< 120	58.9	< 120	83.4	< 120	89.8
	≥ 120	52.5	≥ 120	74.6	≥ 120	80.2
Baseline Urea	< 40	55.1	< 40	77.0	< 40	79.0
	≥ 40	52.5	≥ 40	76.5	≥ 40	68.3
Urea1	< 40	56.6	< 40	84.0	< 40	86.7
	≥ 40	50.9	≥ 40	67.3	≥ 40	76.1
Urea3	< 40	59.7	< 40	86.2	< 40	88.8
	≥ 40	50.3	≥ 40	68.9	≥ 40	77.9
Urea6	< 40	58.4	< 40	93.2	< 40	90.9
	≥ 40	50.0	≥ 40	74.0	≥ 40	81.0
Baseline Creatinine	< 1.5	57.4	< 1.5	78.5	< 1.5	83.8
	≥ 1.5	38.7	≥ 1.5	66.8	≥ 1.5	76.0
Creatinine 1	< 1.5	57.5	< 1.5	79.8	< 1.5	85.1
	≥ 1.5	43.1	≥ 1.5	64.9	≥ 1.5	74.0
Creatinine 3	< 1.5	57.4	< 1.5	82.2	< 1.5	84.9
	≥ 1.5	50.7	≥ 1.5	72.2	≥ 1.5	79.2
Creatinine 6	< 1.5	56.5	< 1.5	76.3	< 1.5	78.6
	≥ 1.5	46.5	≥ 1.5	66.8	≥ 1.5	62.8
Number of BCG doses	< 10	67.2	< 10	68.3	< 10	69.8
	≥ 10	38.3	≥ 10	87.3	≥ 10	90.4

**Fig. 14:**
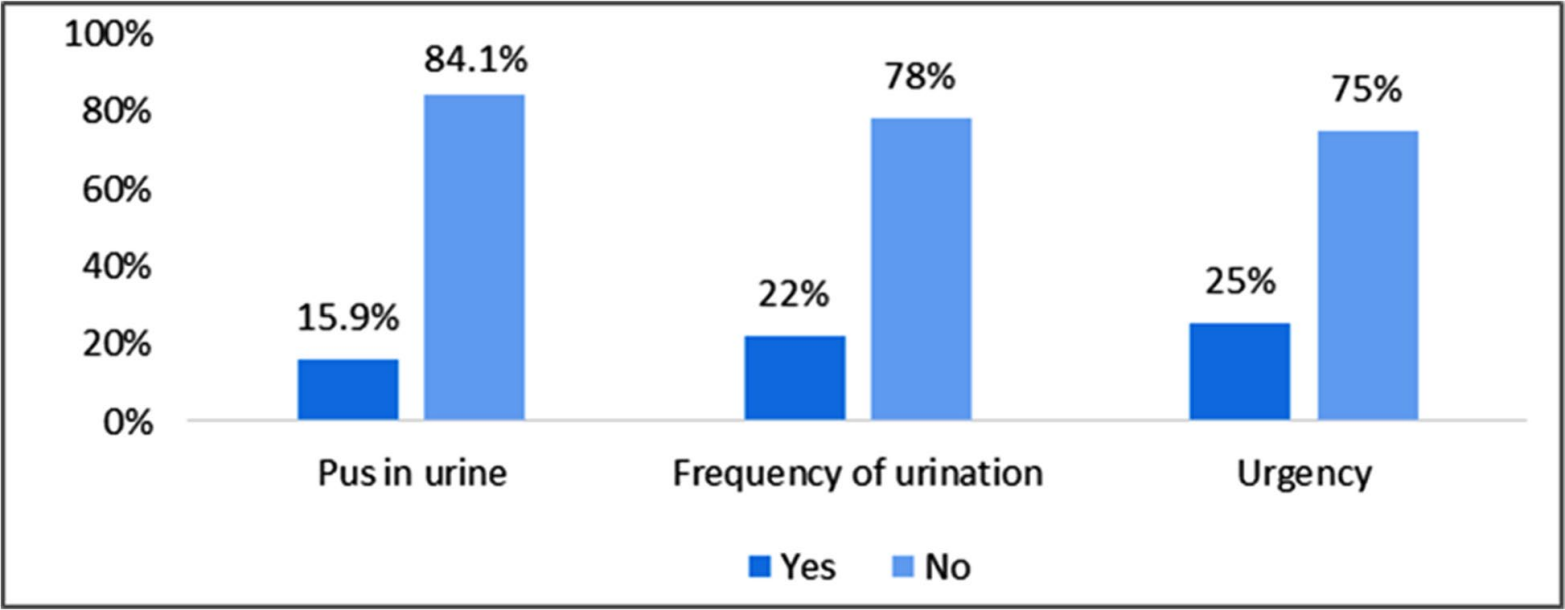
Bar chart representation of the statistically significant adverse events related with BCG dose; represented as percentage (%)

All localized adverse effects were linked to BCG treatment, although only three were statistically significant: frequency of urination, pus in urine, and urgency. Burning sensation was observed in one hundred-five patients (79.5%), seventy-three (55.3%) experienced incontinence, ninety-three (70.5%) experienced hematuria, twenty-nine (22.0%) had increased frequency of urination (P < 0.05), twenty-one (15.9%) experienced pus in urine (P < 0.05), thirty-three (25.0%) experienced increased urgency (P < 0.05) and nineteen (14.4%) experienced increased nocturnal urination (as shown in Table 4 and Fig. 14). Statistical significance indicates that there is a positive relationship between the side effect and the administration of BCG. To attenuate these adverse effects, 95% of patients were prescribed Piperazine-Hexamine-Khellin as an anthelmintic, anti-septic and relaxant and 2 out of 132 patients required further steroid intervention to help reduce the adverse effects of the BCG administration.

**Table 4:** Localized side effects Distribution

Adverse effect	Number	BCG Doses ≤ 10	BCG Doses ≥ 10	Percentage (%)	*p*-value	Progression-free survival (*p*-value)	Recurrence-free survival (*p*-value)	Overall survival (*p*-value)
Burning sensation	105	70	35	79.5%	0.282	0.690	0.376	0.952
Incontinence	73	46	27	55.3%	0.713	0.406	0.367	0.177
Frequency of urination	29	13	19	22.0%	0.013*	0.573	0.569	0.865
Pus in urine	21	9	12	15.9%	0.025*	0.254	0.011*	0.206
Hematuria	93	61	32	70.5%	0.657	0.333	0.232	0.716
Urgency of urination	33	16	17	25.0%	0.028*	0.259	0.797	0.131
Nocturnal urination	19	12	7	14.4%	0.903	0.911	0.861	0.629

**Fig. 15:**
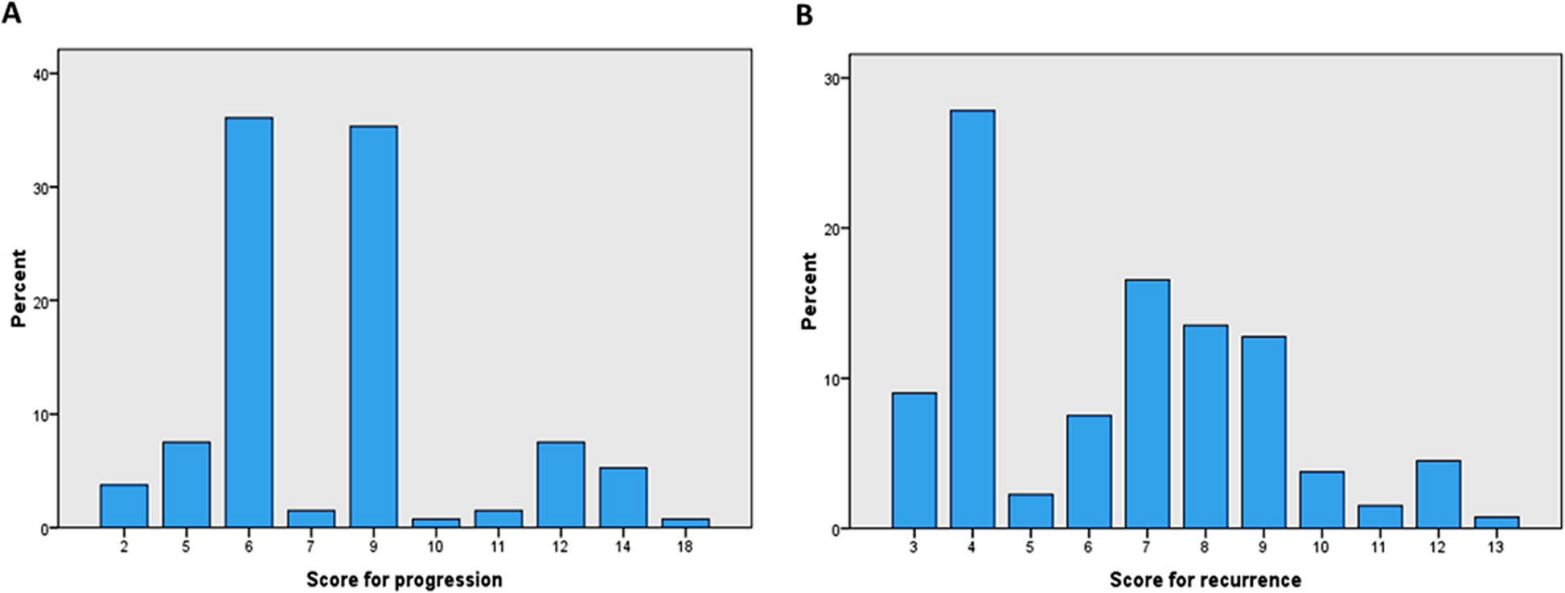
Bar chart representation of the most and least prevalent EORTC progression (**A)** and recurrence (**B**) scores, respectively; among the 132 patients represented as percentage (%)

After calculating the EORTC score of progression and recurrence for the studied cohort, it was observed that approximately 36% and 35% had a score of progression of 6 and 9 respectively, while the least being 0.75% for a score of 10 and 18. As for the score of recurrence, approximately 28% and 17% had a score of 4 and 7, respectively, while the least being 0.75% for a score of 13. As shown in Fig. 15A and B.

**Fig. 16:**
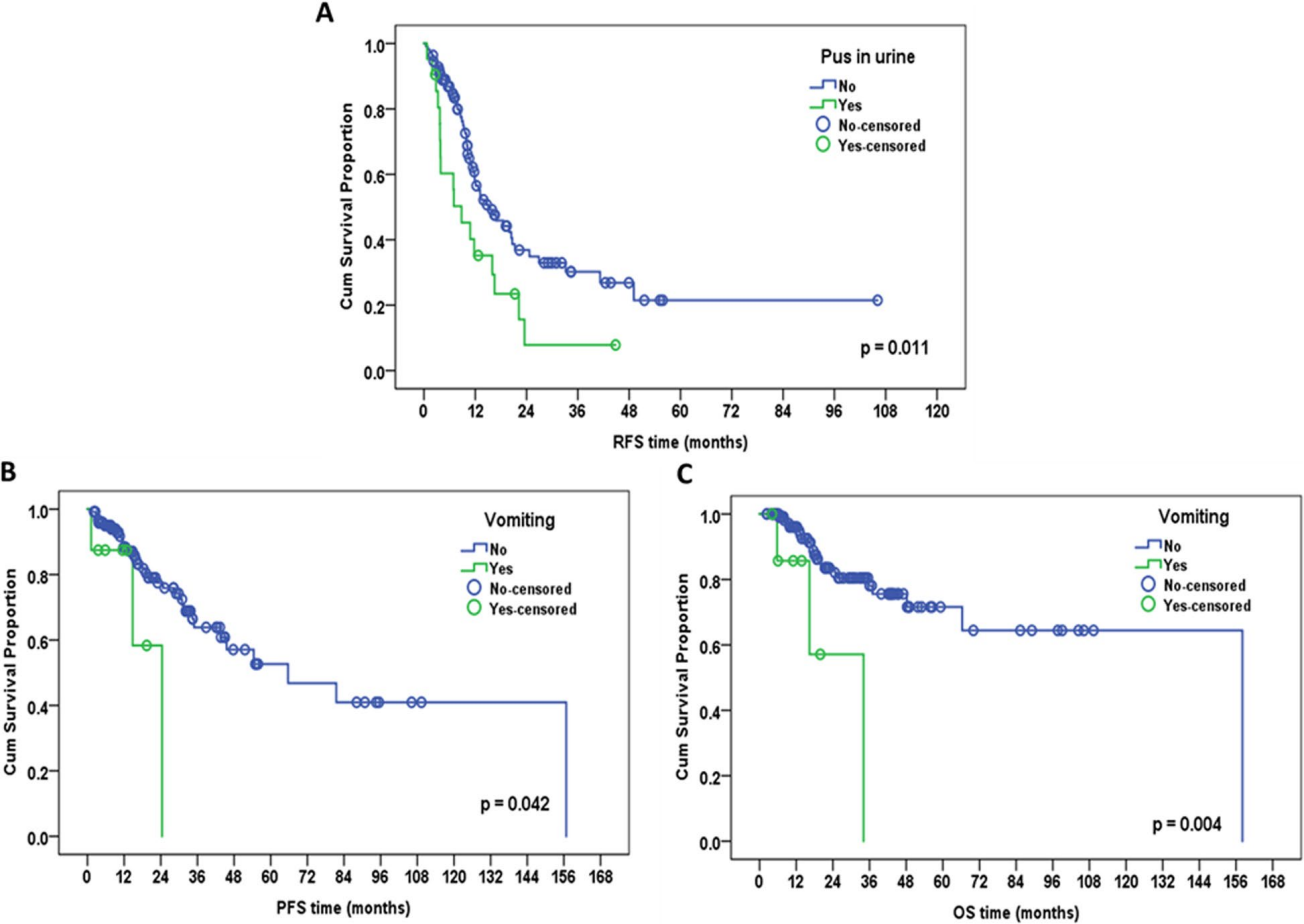
Statistically significant BCG-related side effects; pus in urine (A on RFS), Vomiting (B on PFS and C on OS) impact on survival

The current study demonstrated that all localized adverse effects except for pus in urine had no significant effect on survival. Using Kaplan–Meier analysis to evaluate patients’ recurrence free survival (RFS), it revealed that the presence of pus in urine resulted in a shorter RFS by 6 months. According to the findings of the univariate analysis of RFS, pus in the urine was the only independent factor that had a significant impact on RFS. Individuals who had pus in urine were twice as likely to have a recurrence and had a lower RFS than those who did not. As shown is Fig. 16A (HR: 2.01 [95% CI: 1.16–3.47]), p: 0.011. Nevertheless, a multivariate analysis eliminated any significance of pus in urine. (HR: 1.388 [95%CI: 0.763–2.525]), p: 0.282.

The correlation of systemic side effects together with the administered BCG doses was assessed and revealed that none of the patients experienced tuberculosis (TB)-like symptoms. However, 8 patients (6.1%) reported vomiting and 31 patients (23.5%) experienced fever, Table 5. For systemic side effects none of them showed a significant relation with the BCG doses. Upon evaluating the relationship of PS with the administered number of BCG doses, an insignificant relation was discovered.

**Table 5:** Frequency of systemic side effects

		Count	Percent
TB like symptoms	No	132	100.0%
	Yes	0	0.0%
Vomiting	No	124	93.9%
	Yes	8	6.1%
Fever	No	101	76.5%
	Yes	31	23.5%

Upon studying the impact of vomiting status it showed that patients who suffered vomiting episodes had a shorter PFS of 24 months compared to 66 months in patients who did not experience vomiting. This result was mirrored in OS; where patients who had vomiting events showed a shorter OS of 34 months compared to 158 months in patients who did not experience vomiting (*p* = 0.004), Fig. 16B and C. Univariate analysis PFS showed that patients who experienced vomiting had worse PFS compared to those who did not (HR: 3.3 [95%CI:1.2–11.2), *p* = 0.05. Also, patients who suffered vomiting episodes had worse OS compared to those who had not (HR: 5.1 [95%CI:1.5–17.7), *p* = 0.01. Worth mentioning that univariate analysis of OS; despite of not reaching statistical significance; it showed that patients who had fever had worse OS compared to those who had not (HR: 2.1 [95%CI:0.95–5).

## Discussion

BCG immunotherapy is a standard and unique approach in managing NMIBC cancer. However, its exact mechanisms and the factors influencing its effectiveness and toxicities are subjects of ongoing research [2]. The root causes of the difficulties after instillation of BCG are still unidentified [19]. The optimal clinical usefulness of various cancer treatment modalities is usually hindered by the development of serious complications [20]. Accordingly, the current study aims to investigate the impact of BCG side effects on progression-free survival (PFS), recurrence-free survival (RFS) and overall survival (OS) and bring out the possible adverse effects associated with intravesical BCG. Moreover, to evaluate the impact of adverse reactions on patients’ compliance.

Upon investigating the effect of different sociodemographic and pathological characteristics on the toxicity and efficacy of intravesical BCG in Egyptian patients with non-muscle invasive bladder cancer (NMIBC); it was observed that gender does not impact survival. In harmony, Bree and colleagues (2021) documented that gender does not influence survival on a cohort of 541 patients [21]. Moreover, it is well established that NMIBC is more common in elderly [22, 23], the median age of this study was 60 years. In accordance with gender; age in this study did not impact survival. Previous studies proved that age grouping did not impact survival which was confirmed by this study because it has comparable age group [24]. In contrast, Kohjimoto et al., demonstrated that age was a significant predictor for progression and recurrence at age cutoff of 80 years [25]. This can be justified by the difference in the median age of the studied cohort.

The current observation showed no association between family history and survival. Previously, Egbers and others suggested that families with positive disease history have high level of awareness which helps in early detection and diagnosis. Moreover, the first-degree of NMIBC family history presents a more favorable tumor profile and has better prognosis, but lacking statistical difference between family history and survival [26]. In harmony with the current findings, the authors claimed no strong statistical correlation between family history and prognosis. Regarding occupational hazard, it is considered high risk factor in Egyptian population [27, 28], most of them were exposed to exhaust fumes and chemical compounds [29]. However, the present study aligned with Carta et al. (2018) revealed that occupation hazard does not influence survival. [30]

Regarding comorbidities, no influence was observed on RFS or PFS for hypertensive or diabetic participants. This result is similar to the study by Teleka and colleagues whereby they found no association between blood pressure and NMIBC progression or recurrence [31]. The currently observed absence of association between Diabetes mellitus (DM) and survival was previously documented by various publications [32–34]. In contrast, previous studies proved that DM influenced PFS and RFS [35]; moreover, un-controlled diabetes was correlated with aggressive features of NMIBC as high grade of tumor [36, 37]. Other studies suggested that DM frequently induced urinary tract infection (UTI) [38], which leads to increased bladder cancer risk [39], and frequent exposure to hyperglycemia or hyperinsulinemia increases tumor cell proliferation and metastasis [40], and that increased insulin-like growth factor (IGF)−1 stimulates proliferation and inhibits apoptosis [41]. Additionally, metabolic factors as glucose and IGF-1 decrease production of heparan sulphate proteoglycan; the major constituent of the basement membrane and increase its degradation [39], which leads to change of bladder structure in diabetic NMIBC patients [29]. Herein, reflection of the previous findings was the highly significant positive relation between DM and tumor size; where diabetic NMIBC patients had almost double tumor size compared to non-diabetics.

Considering cardiovascular diseases, a clear negative impact was observed on PFS with P-value of 0.016. Hypoxia is inevitable in cancerous tissues due to its high demand to support continuous cell division [42]. Hypoxic Tumors were proved to possess high metastatic potential; [43] this was attributed to the fundamental metastatic driver, hypoxia inducible factor (HIF) [44]. HIF was documented to favor tumor progression in various cancer types [45] as, breast [46], lung [47], bone [48], prostate [49], colorectal [50], and liver [51]. Recently, HIF has been proven to cause bladder malignancy [52, 53]. Additionally, HIF masters a coping mechanism in cardiovascular diseases and activates defensive responses through regulating its downstream pathway; as vascular endothelial growth factor. [54, 55] Hypoxia inducible factor dual contrasting effect in cardiovascular diseases and cancer might explain the currently observed worse progression free survival in NMIBC patients with cardiac comorbidity.

Herein, grade of tumor impacted OS, but it did not reach statistical significance for PFS and RFS. In contrast, a previous study including 153 patients with NMIBC assessed outcome with two subgroups of grading, high grading group and mixed group, the study proved significant impact of grading on OS, PFS and RFS [56]. Moreover, tumor size had significant influence on RFS, but multi-focality did not impact survival. In contrast, Krajewski and colleagues (2020) confirmed that size and multi-focality significantly affected PFS and RFS. [24] To summarize the currently observed impact of pathological criteria on survival; tumor size influenced RFS and its grade impacted OS. In addition, relation between size of tumor and DM was proved as in other types of malignancies; breast cancer [57] and pancreatic cancer [58].

A study by Green et al. confirmed that, as a result of the intended immune stimulation and cytokine production, minor symptoms following BCG administration are common and usually manageable including fever, malaise, and bladder irritation [59]. Sharma and colleagues investigated 116 patients managed with intravesical BCG and concluded that the most observed adverse effects (26.2%) were frequency of urination, urgency, hematuria; serious side effects mandating treatment interruption and anti-tubercular medications were also reported​ [22]. Matsuoka et al*.* studied 87 NMIBC patients who underwent BGG bladder instillations following TURBT procedure; they concluded that the common adverse events observed were hematuria, pollakiuria, burning sensation, urinary retention, fever, fatigue, edema, and urinary tract infection [60]. While Pérez-Jacoiste and others observed dysuria and frequency in 90% and hematuria in 34% of their studied population​ [61]. In summary, hematuria, urgency and increased urination frequency are acknowledged as the most common side effects. On the contrary, in comparison to the results of the previously stated studies, hematuria (70.5%), burning sensation (79.5%), and incontinence (55.3%) reported in the current study were the most prevalent, regardless of the number of BCG doses.

According to Lentz and Miller (2022), to sustain appropriate lower urinary tract function, the central nervous system as well as the elements of the peripheral nervous system such as somatic and autonomic elements, must work in tandem. Dysfunction arises when these systems are damaged, or when neuromuscular deterioration due to aging or illnesses occurs resulting in urinary incontinence. The TURBT operation itself or false catheterization may be the cause of the harm mentioned above [62]. In agreement, urine incontinence was reported as the most frequently observed adverse effect, affecting 73 out of 132 patients (55.3%). Another issue is the potential for bladder spasms to cause urine to flow from the urethral meatus extrinsic to the catheter [63].​ This clarifies any sense of urgency the patient may have following BCG instillation, which may have contributed to the fact that 25% of the trial participants were unable to keep the intravesical BCG in their bladder for the recommended two hours. To obtain maximal treatment effect, BCG administration technique needs to optimize the duration of the mycobacterium's contact with the bladder urothelium. These measures include limiting the patient's fluid intake before treatment and ensuring total bladder emptying with the use of a lubricated, gently inserted catheter right before BCG instillation while the patient is under gravity [64]. Although similar administration precautions were followed with the currently studied cohort, dysuria affected 79.5%. Lockyer and Gillatt also identified bacterial cystitis and dysuria in 80% of patients [65], which is comparable to the findings of this investigation.​

It is common to find massive pyuria with over 107 WBC/ml of urine within 4–6 h post BCG instillation; this can indicate an inflammation which can be correlated to the mechanism of action of BCG [66]. According to a research by Tan et al*.,* BCG improves macrophage recruitment, differentiation/transformation of the immune system, and macrophage-mediated suppression of bladder cancer development in vivo. [67] As reported by Simons et al., instilling BCG results in localized infection of bladder epithelial cells, which triggers the production of inflammatory cytokines. Furthermore, each round of BCG treatment is followed by a quicker and more persistent inflammatory response, which helps to ensure a positive overall clinical response to BCG immunotherapy [68]. This is supported the current investigation, where the presence of pus in the urine was statistically significant when linked to the administration of BCG; and reflected as a shorter RFS and twice risk for recurrence.

Regarding bleeding; it is common for haematuria to persist after surgery (10–14 days postoperative) [69]. The TURBT treatment has 4–6% complication risk, with urinary tract infections and severe hematuria being the most prevalent, obturator nerve reflex (ONR) and bladder perforation being the most serious consequences [69, 70]. Ensuring optimum penetration to the lesions in the lateral bladder wall during TURBT is an uphill battle. This may cause activation of the nearby obturator nerve, which is situated in close proximity to the wall of the bladder. This stimulation results in ONR, which in turn causes bladder perforation and unintentional bleeding [71]. It is confirmed in 1570 case at a tertiary urology institute that one of the most frequent and serious side effects of TURBT is bladder perforation, which affects about 10% of patients [72]. Also, temporary haematuria is documented in about 26% [64]. In contrast, this study (70.5%) patients experienced haematuria and one out of the 132 patients (0.75%) experienced bladder perforation; this could be attributed not only to the TURBT procedure itself, but also to the difference in surgical experience. Theoretically, learning a surgical procedure takes longer to master it; the complication rate is high and efficacy is lower due to the surgeon's inexperience [73].​

Kim and Patel study also evaluated the possibility of perioperative complications including bladder perforation and ONR; and indicated that the transurethral resection of a bladder tumour is performed utilizing the "incise and scattering" approach, which effectively violates oncological principles by violating surgical margins and increasing the potential of implantation by tumour parts [74]. This might have an impact on oncological outcomes, perhaps increasing the risk of recurrence and progression. Furthermore, Teoh et al*.* stated that TURBT limitations include issues related to determining full removal of the tumour during fractional resection as well as the likelihood of re-implantation of the tumour following surgery. Several recurrence pathways for NMIBC have been documented, including undiscovered tumours during cystoscopy, inadequate resection during TURBT, and tumour re-implantation following TURBT. Furthermore, he suggested that NMIBC's oncological management falls short, citing a 1-year recurrence rate of 15–61% and a 5-year recurrence rate of 31–78% [75]. These recurrence rates were almost mirrored in the current research, with the 1 year and 5 year recurrence rates being almost identical at 60.6% and 39.4%, respectively.

Remarkably, only three of the seven adverse effects that were investigated—pyuria, frequency, and urgency—were statistically significant in relation to the BCG dose administered. In contrast, there was insignificant difference in burning sensation, incontinence, or hemorrhage with BCG doses, thus these side effects might be attributed to the TURBT operation or the erroneous catheterization, or to the BCG administration, justifying the statistical negligibility.

Several systemic side effects were reported among patients receiving BCG therapy; such as fever and vomiting. These findings are consistent with previous research and highlight the immunomodulatory properties of BCG, which can induce an inflammatory response in the body [22]. In this study 14.8% of the population experienced systemic side effects following BCG doses. This findings contrast with the results reported in another study where 31% of the population experienced such side effects [22]. The incidence and severity of systemic side effects varied among patients. Some individuals experienced mild symptoms that resolved without intervention, while others required medical management. In a study by Pérez-Jacoiste and colleagues, it was reported that a systemic adverse event following BCG instillation is characterized as a specific organ infection post BCG instillation that responds to antituberculosis treatment and lacks an alternative diagnosis [61]. Pommier et al. defined BCG infection as the presence of fever lasting at least 48 h without any other identifiable cause, and/or involvement of at least one organ other than the bladder, leading to the discontinuation of BCG therapy. The determination of organ involvement is based on clinical, biological, and radiological abnormalities [76]. Systemic complications are well defined as the presence of fever, chills, hypotension, and progressive multisystem organ failure [74]. These various definitions highlights the diverse and intricate nature of complications arising from BCG immunotherapy.

Regarding the systematic side effects, this study examined the occurrence of tuberculosis-like symptoms, vomiting, and fever among the patients. Notably, none of the patients experienced tuberculosis-like symptoms, which can be attributed to the mass vaccination program implemented in Egypt [77]. Current findings revealed that 23.5% of patients experienced fever, whereas another study reported a lower fever incidence of only 2.9%. It is possible that the disparity in fever rates could be attributed to the difference in treatment completion rates between the two studies. Herein, a higher proportion (92.5%) of patients completed the planned course of BCG doses, whereas in the other study, only 64% of the total patients completed the treatment [22]. Present observation showed that 6% of patients experienced vomiting, whereas another study reported a higher incidence of 21% [71]; which can also be attributed to the mass vaccination program implemented in Egypt [78]. It also revealed that patients who had vomiting episodes had shorter PFS and OS by 42 and 124 months, respectively compared to patients who had not vomiting.

To extend the impact of BCG side effects on patient’s physical well-being, correlation between performance status (PS) before and after BCG doses was performed results revealed that there was a decline in patient’s PS after taking the BCG, this was clearly reflected in the survival analysis. In agreement with the present observation intravesical BCG administration was previously reported as tolerable despite of its induced localized and generalized adverse effects. Moreover, it was documented to decline patient’s physical performance [22].

ALP presence in serum is associated with bone, and liver diseases [79] and its abnormal expression has been linked to a number of human malignancies [80]. This study revealed an elevation in ALP levels after the 1 st, 3rd, and 6th doses of BCG immunotherapy that needs careful consideration. BCG's interaction with the immune system may induce inflammatory responses, affecting liver enzymes. Previous studies documented that elevation of ALP is an independent risk factor for bladder and gastric cancer as well as bone metastases [81, 82]. Moreover, in clear cell chondrosarcoma of the bone, bony metastatic breast as well as prostate cancer, both the baseline and changes in ALP have been identified as predictive variables for therapy impact and survival [78, 83, 84].

The currently observed correlation between elevated ALP levels above 120 IU/L and diminished overall survival (OS) in NMIBC patients taking BCG immunotherapy can be attributed to several possible reasons. Elevated ALP is commonly associated with liver and bone metastases, suggesting a more advanced stage of disease [85]. Bladder cancer patients with higher ALP levels at baseline and during treatment may harbor more aggressive tumors, potentially leading to poorer outcomes [86, 87]. Furthermore, BCG immunotherapy relies on the activation of the immune system to target cancer cells [88], and an elevated ALP might indicate a compromised immune response or a less favorable microenvironment for immunotherapy efficacy. Monitoring ALP levels after each BCG dose becomes essential in assessing treatment response and adapting management strategies based on the evolving disease dynamics. Hence, the correlation between ALP levels and OS underscores the importance of integrating biochemical markers into the comprehensive evaluation of bladder cancer patients undergoing BCG immunotherapy. Previous studies proved that elevated serum ALP is associated with poor OS and PFS in prostate cancer and reported that ALP is a trustworthy marker for prognosis [89, 90]. This was confirmed in a metastatic prostatic cancer Egyptian cohort [91]. In harmony, this study found that the hazard ratio for death was 3.307 (1.105–9.900) at; where patients with higher baseline level (> 120 IU/L) showed shorter OS by 27.4 months.

Simultaneously, elevated levels of urea and creatinine further intensify the complexity of the scenario. These markers, indicative of renal function, may suggest a potential impact on the kidneys during BCG treatment [92]. The immune response triggered by BCG, while targeting cancer cells, might inadvertently affect renal function [93]. Recently, elevated levels of urea and creatinine were used for prognosis of risk classification in more than one thousand patient with prostate malignancy; authors concluded the independency of creatinine level as a predictor of prognosis and that its control may have a favorable impact on patients outcome [94]. Regular monitoring and understanding the temporal pattern of these elevations after each dose are crucial for assessing treatment-related effects versus underlying pathology. The current study showed a notable association of urea and creatinine levels with OS; elevated urea and creatinine often indicates impaired renal function, potentially linked to BCG-induced nephrotoxicity [95]. The compromised renal function may contribute to poorer outcomes due to impaired drug metabolism and potential systemic effects [96]. Monitoring urea and creatinine levels at baseline and after subsequent BCG doses becomes crucial in identifying patients at risk of renal complications, allowing for timely intervention and dose adjustments. The observed correlation highlights the importance of renal function in predicting outcomes during BCG immunotherapy, emphasizing the need for vigilant monitoring and tailored management strategies for individuals with higher urea and creatinine levels. This pattern of biomarker elevation raises questions about the need for closer renal and hepatic monitoring during BCG immunotherapy [97]. Collaborative efforts between oncologists, nephrologists, and hepatologists could shed light on the intricate dynamics and guide strategies to mitigate potential adverse effects while optimizing the therapeutic benefits of BCG in bladder cancer management.

This study showed lower RFS with statistical significance for above a total of 10 BCG doses. This could be attributed to several factors; higher number of doses may trigger adverse effects or immune system saturation, leading to diminished therapeutic effects. Additionally, excessive doses might induce immune tolerance or alter the balance of the immune response, influencing the treatment's overall effectiveness. Previous *in-vitro* studies reported that tumor cells in bladder are susceptible to the direct cytotoxic effects of BCG [98–100]. It is well-established that BCG-treated patients have more macrophages in their urine and bladder walls than untreated patients [101, 102]. Remarkably, higher concentrations of CD68 + CD163 + macrophages, often known as "M2" macrophages, are associated with an increased risk of cancer recurrence following BCG therapy, indicating that these macrophages may inhibit the immune response triggered by BCG [103, 104]. Further investigations into the immunological mechanisms and potential dose–response relationships are necessary to pinpoint the precise reasons behind this observation. The improved PFS and OS in NMIBC patients receiving 10 or more BCG doses compared to those with fewer than 10 doses might be credited to the enhanced immune response and prolonged immunomodulatory effects of BCG therapy. A higher number of BCG doses likely ensures sustained activation and recruitment of immune cells, particularly T lymphocytes, which play a crucial role in recognizing and eliminating cancer cells [105]. This prolonged immune-stimulatory environment created by a sufficient BCG dosage could result in better control of tumor growth and micro-metastases, reduced recurrence, and ultimately improved PFS [106, 107]. Consequently, optimizing BCG dosing regimens is crucial in maximizing therapeutic outcomes for NMIBC patients.

The current study was carried out at National Cancer Institution (NCI), Cairo University, which receives heterogeneous patient population from all over Egypt, of different sociodemographic backgrounds; compensating the partially small sample size. Although confined to one hospital the population could be considered representative to the Egyptian NMIBC patients. Being a retrospective study makes it prone to recall and documentation bias. Thus, authors recommend further prospective multi-centric investigation on a larger cohort to enhance its external validity, providing a more comprehensive understanding of the factors under investigation. This approach would not only strengthen the robustness of the current findings, but also contribute significantly to the advancement of knowledge in NMIBC.

## Conclusion

This study highlights the critical significance of Bacillus Calmette-Guerin (BCG)-induced side effects on patient outcomes and provides insight on the complex landscape of NMIBC therapy. Adverse effects are either specific or non-specific to BCG, the non-specific risks relate to the TURBT procedure or urethral catheterization before BCG instillation. The nuanced analysis of adverse reactions reveals intriguing insights, with notable significance found in the association between pyuria, revealing that the presence of pus in urine resulted in a shorter RFS by 6 months. Clear decline of patients’ performance status was attributed to BCG therapy; and reflected as worse survival for PS 4. Tuberculosis-like symptoms was undetected in the currently studied cohort due to the mass vaccination program implemented in Egypt, but vomiting was recorded in 6.1% and fever in 23.5% with an observed negative impact on survival. Moreover, cardiac comorbidity as well as tumor grade and size in NMIBC patients affects survival and might be evaluated as independent prognostic factors for Egyptian NMIBC patients. Clear relation between DM and tumor size was observed in the studied cohort; where larger tumor size was reflected as shorter RFS.

The study provided compelling insights into the patterns of elevation in ALP, urea, and creatinine levels across multiple BCG therapy cycles in Egyptian patients with NMIBC. Notably, a discernible correlation emerged, indicating that ALP levels exceeding 120 were associated with adverse impacts on survival. This emphasizes the clinical significance of monitoring ALP as a potential prognostic marker during BCG therapy. It also revealed a noteworthy trend regarding the number of BCG doses administered. Patients receiving more than 10 doses exhibited lower recurrence-free survival compared to those with fewer than 10 doses. This observation prompts a critical evaluation of the optimal dosage strategy for NMIBC management, suggesting that a judicious approach to BCG dosing may contribute to improved patient outcomes. Collectively, these findings accentuate the importance of vigilant monitoring and personalized dosage strategies in BCG therapy for NMIBC, offering valuable insights for refining clinical protocols and enhancing the overall efficacy of this treatment modality.

## Supplementary Information

Supplementary Material 1.

## Data Availability

No datasets were generated or analysed during the current study.
